# RAS–PI3Kα signaling regulates Kras^G12D^-induced lymphangiogenesis in complex lymphatic anomalies

**DOI:** 10.1242/dmm.052932

**Published:** 2026-08-27

**Authors:** Lorenzo M. Fernandes, Jeffrey Tresemer, Angelica Vallejo, Alice Yao, Joshua P. Scallan, Michael T. Dellinger

**Affiliations:** ^1^ Hamon Center for Therapeutic Oncology Research, UT Southwestern Medical Center, Dallas, TX 75390, USA; ^2^ Department of Molecular Pharmacology and Physiology, Morsani College of Medicine, University of South Florida, Tampa, FL 33612, USA; ^3^ Department of Surgery, UT Southwestern Medical Center, Dallas, TX 75390, USA

**Keywords:** Complex lymphatic anomaly, KRAS, *PIK3CA*, Lymphangiogenesis, Lymphatic malformation, Gorham–Stout disease, Generalized lymphatic anomaly, Kaposiform lymphangiomatosis, Central conducting lymphatic anomaly

## Abstract

Complex lymphatic anomalies (CLAs) are rare diseases characterized by the abnormal development of lymphatic vessels. CLAs can be caused by somatic activating variants in *KRAS* (e.g. KRAS^G12D^), which stimulate MAPK and PI3K signaling. Although KRAS–MAPK signaling is known to play a critical role in CLA pathogenesis, the contribution of KRAS–PI3Kα signaling remains unclear. To investigate the role of RAS activation of PI3Kα in CLAs, we analyzed mice carrying two missense mutations in the RAS-binding domain of p110α (encoded by *Pik3ca*), the catalytic subunit of PI3Kα. These two mutations block the interaction between p110α and RAS but do not affect its kinase activity. Disruption of RAS-mediated PI3Kα activation in lymphatic endothelial cells reduced lymphatic vessel branching but did not affect lymphatic valve formation. In Kras^G12D^-mutant mice, blocking RAS activation of p110α reduced the pathological enlargement of lymphatic vessels, but failed to prevent Kras^G12D^-induced lymphatic valve loss. Similar results were observed following p110α deletion in Kras^G12D^-mutant mice. Together, these findings demonstrate that Kras^G12D^ drives distinct disease phenotypes through separate downstream pathways. KRAS–PI3Kα signaling promotes pathological lymphatic vessel enlargement, whereas KRAS–MAPK signaling disrupts lymphatic valve formation.

## Teaser

**Summary:** The somatic activating Kras^G12D^ variant associated with complex lymphatic anomalies drives distinct phenotypes through separate pathways – KRAS–PI3Kα signaling promotes lymphatic vessel enlargement, whereas KRAS–MAPK signaling disrupts lymphatic valve formation.

## INTRODUCTION

Complex lymphatic anomalies (CLA) are rare diseases caused by errors in lymphatic vessel development (Trenor and Chaudry, 2014). CLA is an umbrella term that covers Gorham–Stout disease, kaposiform lymphangiomatosis, generalized lymphatic anomaly and central conducting lymphatic anomaly (Makinen et al., 2021). Patients with CLAs exhibit a spectrum of phenotypes, including dilated lymphatic vessels, diffuse lymphatic malformations, chylous ascites, chylothorax, lymphedema and ectopic lymphatic vessels in bone (Makinen et al., 2021). Somatic activating variants in *KRAS* have recently been found to cause some cases of CLAs (Andreoti et al., 2023; Homayun-Sepehr et al., 2021; Li et al., 2019, 2023; Nozawa et al., 2020; Sheppard et al., 2023). KRAS is a small G protein that induces signaling when bound to GTP and is inactive when bound to GDP (Haigis, 2017). The *KRAS* variants that cause CLAs impair GTP hydrolysis or increase GDP/GTP exchange, resulting in hyperactive KRAS signaling (Haigis, 2017). Interestingly, the same *KRAS* variants that cause CLAs also cause cancer (e.g. pancreatic ductal carcinoma, lung adenocarcinoma), and the treatment of CLAs has benefited greatly from pharmacological advances in treating RAS pathway-driven cancers (Chowers et al., 2023; Foster et al., 2020; Haigis, 2017; Li et al., 2019, 2023).

Animal models have helped uncover the effects of hyperactive KRAS signaling on the development of lymphatic vessels. Zebrafish embryos expressing active forms of KRAS in the lymphatic endothelium display edema and enlarged lymphatic vessels (Sheppard et al., 2023). Similarly, mouse embryos that express Kras^G12D^ in their lymphatic endothelial cells (LECs) exhibit edema, dilated lymphatic vessels and lymphovenous valve defects (Fernandes et al., 2023). Neonatal expression of Kras^G12D^ in LECs stimulates lymphatic vessel invasion of bone, a pathological process frequently seen in CLAs (Homayun-Sepehr et al., 2021). It also suppresses the development of lymphatic valves and the recruitment of lymphatic muscle cells to lymphatic vessels (Homayun-Sepehr et al., 2021; Mastrogiacomo et al., 2026). We recently used single-cell RNA sequencing (scRNA-seq) to investigate the effect of Kras^G12D^ on the cellular and transcriptional landscape of lymphatic vessels (Fernandes et al., 2025). We found that the cellular profile of lymphatic vessels of adolescent Kras^G12D^-mutant mice was similar to that of newborn control mice (Fernandes et al., 2025). We also found that immature lymphatic vessels are more sensitive to the pathologic effects of Kras^G12D^ than mature lymphatic vessels (Fernandes et al., 2025). Collectively, these results suggest that hyperactive KRAS signaling impairs the remodeling and maturation of lymphatic vessels.

The mitogen-activated protein kinase (MAPK) pathway is a well-known signaling pathway activated by KRAS. Trametinib is a US Food and Drug Administration (FDA)-approved inhibitor of the MAPK kinases MEK1 and MEK2 (hereafter MEK1/2), which is used to block MAPK signaling and is an emerging medical therapy for CLAs (Seront et al., 2024). Patients with CLA treated with trametinib show improved lung function, decreased effusions and altered lymph drainage patterns (Chowers et al., 2023; Li et al., 2019). We have found that trametinib reduces MAPK activation and increases the expression of lymphatic valve genes in Kras^G12D^ LECs (Fernandes et al., 2023). We also reported that trametinib suppresses the loss of lymphatic valves in Kras^G12D^-mutant mice (Homayun-Sepehr et al., 2021). Additionally, MEK1/2 inhibition decreases the incidence of edema in a KRAS^G12D^-mutant zebrafish model of CLA (Sheppard et al., 2023). Although these reports establish that the MAPK pathway is critical in the pathogenesis of KRAS-induced CLAs, the function of other downstream pathways in KRAS-driven CLAs remains unclear.

Phosphoinositide 3-kinase (PI3K) is an important downstream effector of KRAS. PI3K phosphorylates phosphatidylinositol-4,5-bisphosphate to create phosphatidylinositol-3,4,5-trisphosphate, which induces signaling by interacting with lipid-binding proteins (Fruman et al., 2017). Class 1A PI3Ks function as heterodimers composed of a catalytic subunit [p110α (encoded by *PIK3CA*), p110β (*PIK3CB*) or p110δ (*PIK3CD*)] and a regulatory subunit [p85α, p55α, p50α (splice variants of *PIK3R1*), p85β (*PIK3R2*) or p55γ (*PIK3R3*)] (Graupera and Potente, 2013). Under basal conditions, the regulatory subunit of PI3K inhibits the catalytic subunit of PI3K. However, this inhibition is relieved upon recruitment of the heterodimer to activated receptor tyrosine kinases (Graupera and Potente, 2013). Additionally, RAS proteins work synergistically with receptor tyrosine kinases to recruit PI3Kα (and PI3Kδ) to the plasma membrane (Buckles et al., 2017; Rodriguez-Viciana et al., 1996; Siempelkamp et al., 2017). In recent years, there has been a growing appreciation that alterations in PI3K signaling impact the development of lymphatic vessels. Somatic hotspot variants in *PIK3CA* (gene encoding p110α) have been found in patients with cystic lymphatic malformations and CLAs (Boscolo et al., 2015; Grenier et al., 2023; Li et al., 2023; Luks et al., 2015; Osborn et al., 2015; Rodriguez-Laguna et al., 2019). These variants cause excessive PI3Kα signaling in LECs and defects in the morphogenesis of lymphatic vessels (Martinez-Corral et al., 2020; Petkova et al., 2023; Rodriguez-Laguna et al., 2019). Conversely, conditional knockout of *Pik3ca* in LECs impairs the development of mesenteric lymphatic vessels in mouse embryos and the development and maturation of lymphatic vessels in the skin of newborn mice (Korhonen et al., 2022; Stanczuk et al., 2015). These studies show that PI3Kα signaling must be tightly regulated for lymphatic vessels to develop and remodel properly.

The role of KRAS–PI3Kα signaling in the development and progression of CLAs is poorly understood. To specifically study the role of RAS activation of p110α in development and disease, mice were created that carry two germline missense mutations (p.T208D, p.K227A) in the RAS-binding domain (RBD) of *Pik3ca* (herein referred to as *Pik3ca^RBD^*) (Gupta et al., 2007). These two mutations block the interaction between p110α and RAS but do not affect its catalytic activity or interaction with p85 (Gupta et al., 2007). Importantly, specifically blocking RAS activation of p110α impairs tumor angiogenesis and EGFR- and KRAS-driven tumor development and maintenance (Castellano et al., 2013; Gupta et al., 2007; Murillo et al., 2018, 2014). Surprisingly, *Pik3ca^RBD/RBD^* mice exhibit lymphatic vessel defects (Gupta et al., 2007). Most *Pik3ca^RBD/RBD^* mice die during embryonic development (Gupta et al., 2007). However, *Pik3ca^RBD/RBD^* mice that survive embryonic development display chylous ascites and submucosal edema (Gupta et al., 2007). Additionally, *Pik3ca^RBD/RBD^* embryos have fewer lymphatic vessels in the skin, diaphragm and chest wall than control embryos (Gupta et al., 2007). Because RAS activation of p110α is blocked in all cells in *Pik3ca^RBD/RBD^* mice, it is unclear whether the abnormal lymphatic vessel development in *Pik3ca^RBD/RBD^* mice is due to cell-autonomous or non-cell-autonomous effects. In the present study, we use the *Pik3ca^RBD^* strain and Cre-loxP system to identify the cell-autonomous role of RAS–p110α signaling in lymphatic vessel development and to characterize the effect of blocking the interaction between RAS and p110α on Kras^G12D^-induced lymphatic malformations.

## RESULTS

### Expression pattern of class 1 PI3K isoforms and RAS genes by LECs

**Fig. 1.**
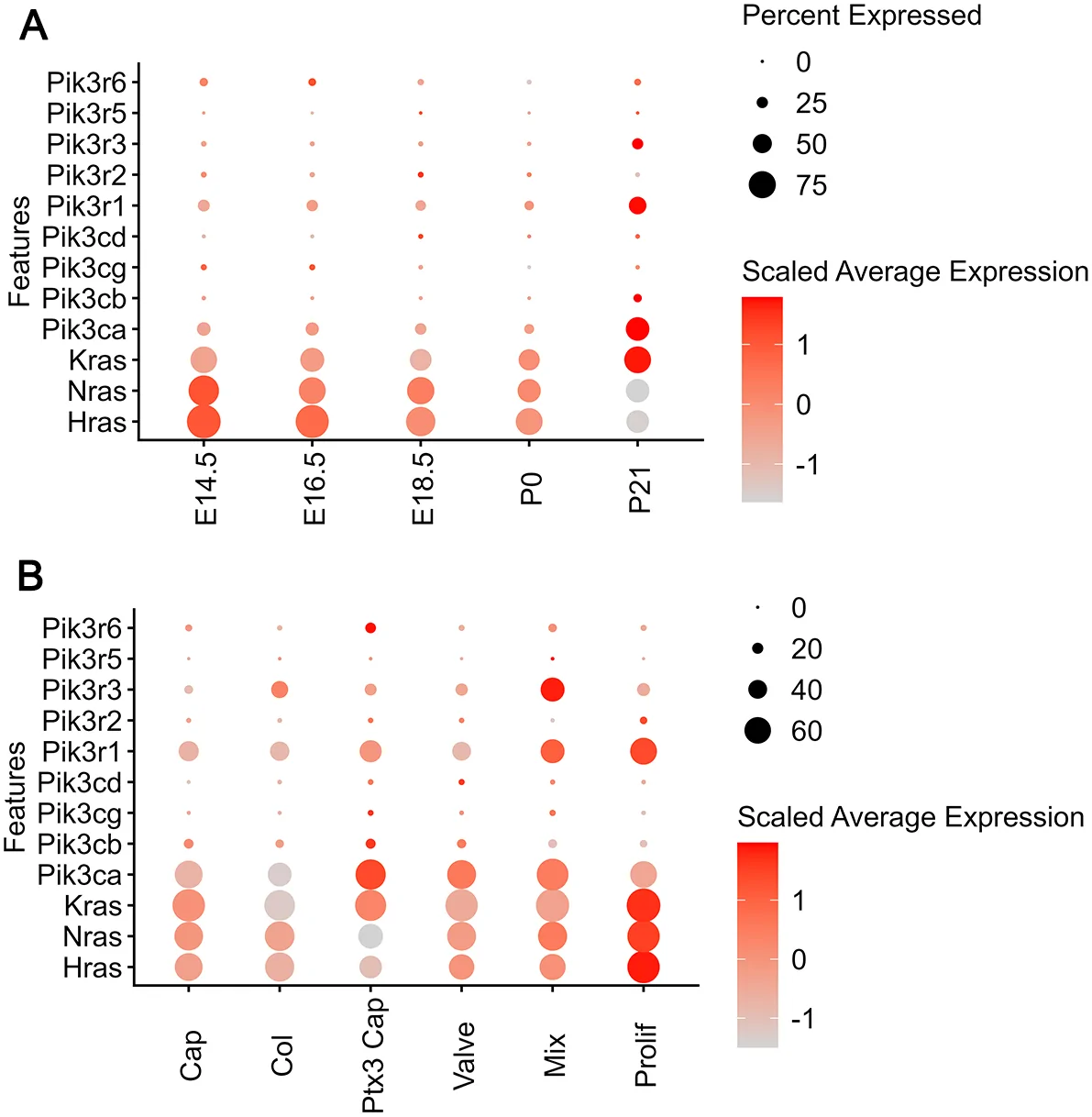
**The expression pattern of PI3K class 1 isoforms and common RAS genes in LECs as revealed by scRNA-seq.** (A) A dot plot illustrating the expression levels of selected genes in single-cell RNA-sequencing (scRNA-seq) data for lymphatic endothelial cells (LECs) at different developmental stages: embryonic day (E) 14.5, E16.5, E18.5, postnatal day (P) 0 and P21. (B) A dot plot showing the expression of select genes by LEC subtypes at P21. Cap, capillary; Col, collecting; Prolif, proliferating.

To investigate the relationship between RAS and PI3K in LECs, we analyzed the expression of class 1 PI3K catalytic subunits (encoded by *Pik3ca*, *Pik3cb*, *Pik3cg*, and *Pik3cd*) and regulatory subunits (encoded by *Pik3r1*, *Pik3r2*, *Pik3r3*, *Pik3r5*, *Pik3r6*), as well as common RAS genes (*Hras*, *Nras* and *Kras*) in published scRNA-seq datasets of lung LECs that span five time points [embryonic day (E) 14.5, E16.5, E18.5, postnatal day (P) 0 (GSE213744; Fu et al., 2023) and P21 (Fernandes et al., 2025)]. This analysis revealed that *Pik3ca* is the gene representing the most highly expressed catalytic subunit in LECs, and that *Pik3r1* is the gene representing the most highly expressed regulatory subunit (Fig. 1A). Interestingly, *Pik3ca* and *Kras* expression in LECs increased during development (Fig. 1A). In contrast, the expression of *Hras* and *Nras* in LECs decreased over time (Fig. 1A). We then surveyed the expression of these genes by the different subtypes of LECs in the dataset for P21 mice. We previously identified six distinct subtypes of lymphatic endothelium in 21-day-old mice (Fernandes et al., 2025). These subtypes include Ptx3, capillary, collecting, valve and proliferating LECs. There is also a subtype called ‘mix’, which expresses markers of Ptx3 and collecting LECs. We found that all subtypes expressed *Pik3ca*, *Pik3r1*, *Hras*, *Nras* and *Kras* (Fig. 1B). However, *Pik3ca* expression was highest in Ptx3 LECs, whereas the expression of *Hras*, *Nras* and *Kras* was greatest in proliferating LECs (Fig. 1B). Next, we examined the expression of class 1 PI3K catalytic and regulatory subunits in human LECs using publicly available bulk RNA-seq data (GSE239737; Fernandes et al., 2023). Like in the mouse, we found that *PIK3CA* and *PIK3R1* were the genes representing the most highly expressed PI3K catalytic and regulatory subunits, respectively (Fig. S1).

### Lymphatic development is normal in *Pik3ca^wt/RBD^* mice

To characterize the role of RAS activation of p110α in lymphatic vessel development, we obtained *Pik3ca^RBD^* mice. We bred *Pik3ca^wt/RBD^* mice together to generate *Pik3ca^wt/wt^*, *Pik3ca^wt/RBD^* and *Pik3ca^RBD/RBD^* offspring. We were unable to obtain *Pik3ca^RBD/RBD^* mice that survived at least 7 days after birth, suggesting that *Pik3ca^RBD/RBD^* mice exhibit embryonic or neonatal lethality (Table 1). To determine whether *Pik3ca^wt/RBD^* mice exhibit lymphatic vessel defects, we analyzed ear skin from 3-week-old mice by whole-mount immunofluorescence staining (Fig. S2A). We found that the number of lymphatic branch points was not significantly different between *Pik3ca^wt/wt^* and *Pik3ca^wt/RBD^* mice (Fig. S2B). However, the diameter of lymphatic vessels was slightly smaller in *Pik3ca^wt/RBD^* mice compared to that in *Pik3ca^wt/wt^* mice (Fig. S2C). We also examined lymphatic valves and found that the number of lymphatic valves was not significantly different between *Pik3ca^wt/wt^* and *Pik3ca^wt/RBD^* mice (Figs S2D,E). These results suggest that lymphatic development is relatively unaffected in *Pik3ca^wt/RBD^* mice.

**Table 1.** Genotyping results for offspring obtained from crosses between *Pik3ca^wt/RBD^* mice.

Genotype	Observed	Expected
*Pik3ca^wt/wt^*	32	18
*Pik3ca^wt/RBD^*	41	37
*Pik3ca^RBD/RBD^*	0	18

### Lymphatic branching is reduced in *Pik3ca^RBD/RBD^* embryos

**Fig. 2.**
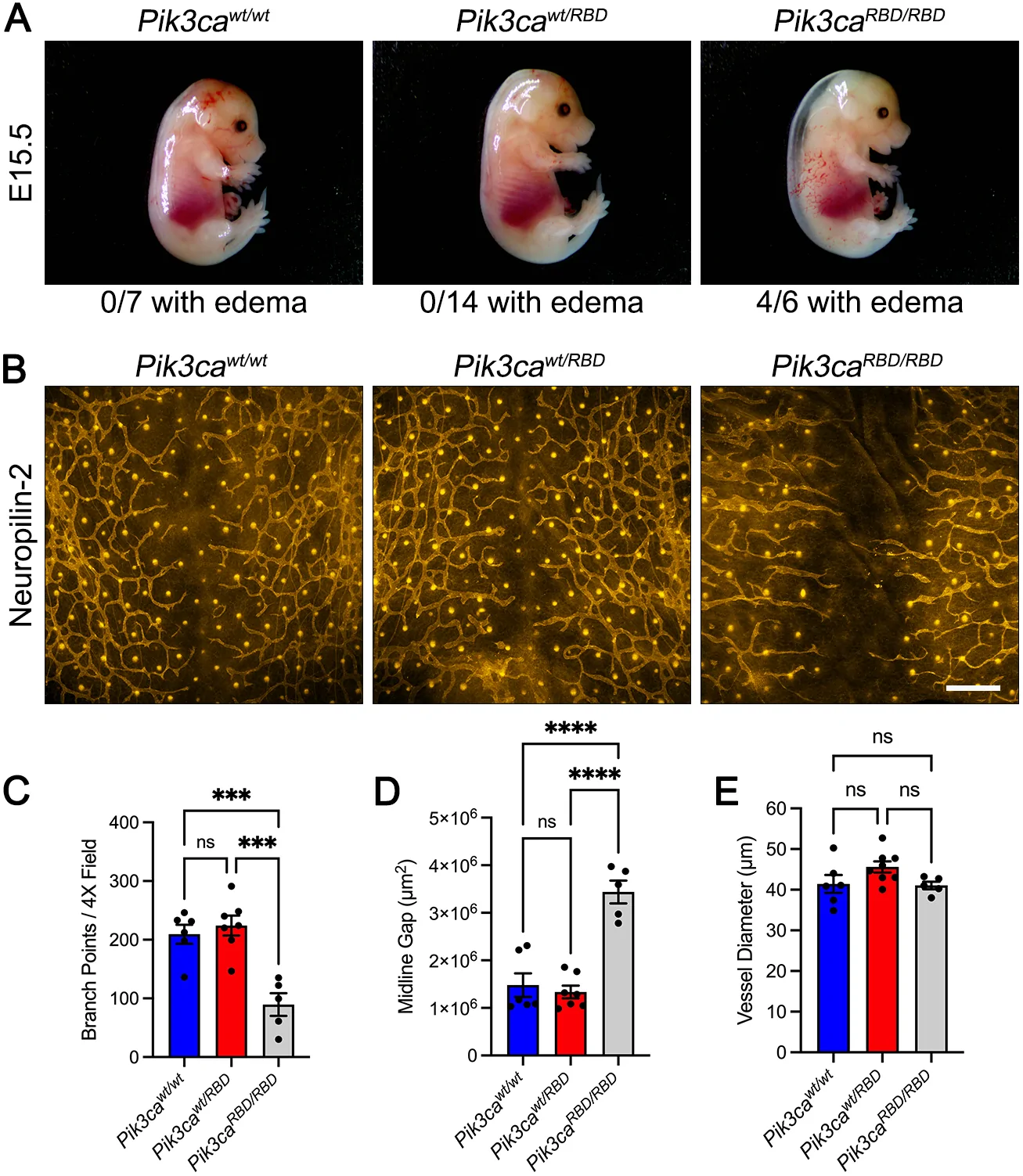
**Lymphatic vessel development is impaired in *Pik3ca^RBD/RBD^* embryos.** (A) Representative images of E15.5 embryos. Four out of six *Pik3ca^RBD/RBD^* embryos had edema. (B) Representative images of back skin from E15.5 embryos immunostained for neuropilin-2. Scale bar: 500 μm. (C) Lymphatic branch points for *Pik3ca^wt/wt^* (209.4±16.22; *n*=6), *Pik3ca^wt/RBD^* (224.1±16.86; *n*=7) and *Pik3ca^RBD/RBD^* (89.50±19.47; *n*=5) embryos. (D) Midline gap values for *Pik3ca^wt/wt^* [(1.48±0.25)×10^6^ μm^2^; *n*=6], *Pik3ca^wt/RBD^* [(1.34±0.13)×10^6^ μm^2^; *n*=7] and *Pik3ca^RBD/RBD^* [(3.44±0.24)×10^6^ μm^2^; *n*=5] embryos. (E) Lymphatic diameters for *Pik3ca^wt/wt^* (41.42±2.19 μm; *n*=6), *Pik3ca^wt/RBD^* (45.61±1.37 μm; *n*=8) and *Pik3ca^RBD/RBD^* embryos (41.06±0.93 μm; *n*=5). Data are presented as mean±s.e.m. *n*=number of mice. Ns, not significant; ***P<0.001; ****P<0.0001; one-way ANOVA with Tukey's multiple comparisons (C,E) and Kruskal–Wallis nonparametric test with Dunn's multiple comparisons test (D).

Next, we examined *Pik3ca^RBD/RBD^* embryos to determine whether lymphatic development was altered in mice lacking RAS activation of p110α. After breeding *Pik3ca^wt/RBD^* mice together, we found that *Pik3ca^RBD/RBD^* mice were at their expected Mendelian ratio on E15.5 (Table 1). In contrast to *Pik3ca^wt/wt^* and *Pik3ca^wt/RBD^* embryos, *Pik3ca^RBD/RBD^* mice displayed edema, which is suggestive of a lymphatic defect (Fig. 2A). We then analyzed lymphatic vessels in the back skin of E15.5 embryos by whole-mount immunofluorescence staining for neuropilin-2 (NRP2) (Fig. 2B). We found that *Pik3ca^RBD/RBD^* embryos had fewer lymphatic branch points than *Pik3ca^wt/wt^* and *Pik3ca^wt/RBD^* embryos (Fig. 2C). Additionally, the midline lymphatic gap was significantly greater in *Pik3ca^RBD/RBD^* embryos than in *Pik3ca^wt/wt^* and *Pik3ca^wt/RBD^* embryos, suggesting that RAS activation of p110α regulates lymphatic sprouting (Fig. 2D). Despite the changes in lymphatic vessel density, lymphatic vessel diameters were not significantly different between the embryos (Fig. 2E). Our results demonstrate that blocking RAS activation of p110α impairs the development of lymphatic vessels. However, it was unclear whether the effects of the *Pik3ca^RBD^* mutation on lymphatic development were due to cell-autonomous or non-cell-autonomous effects.

### Blocking the interaction between RAS and p110α in LECs affects lymphatic branching but not lymphatic remodeling

**Fig. 3.**
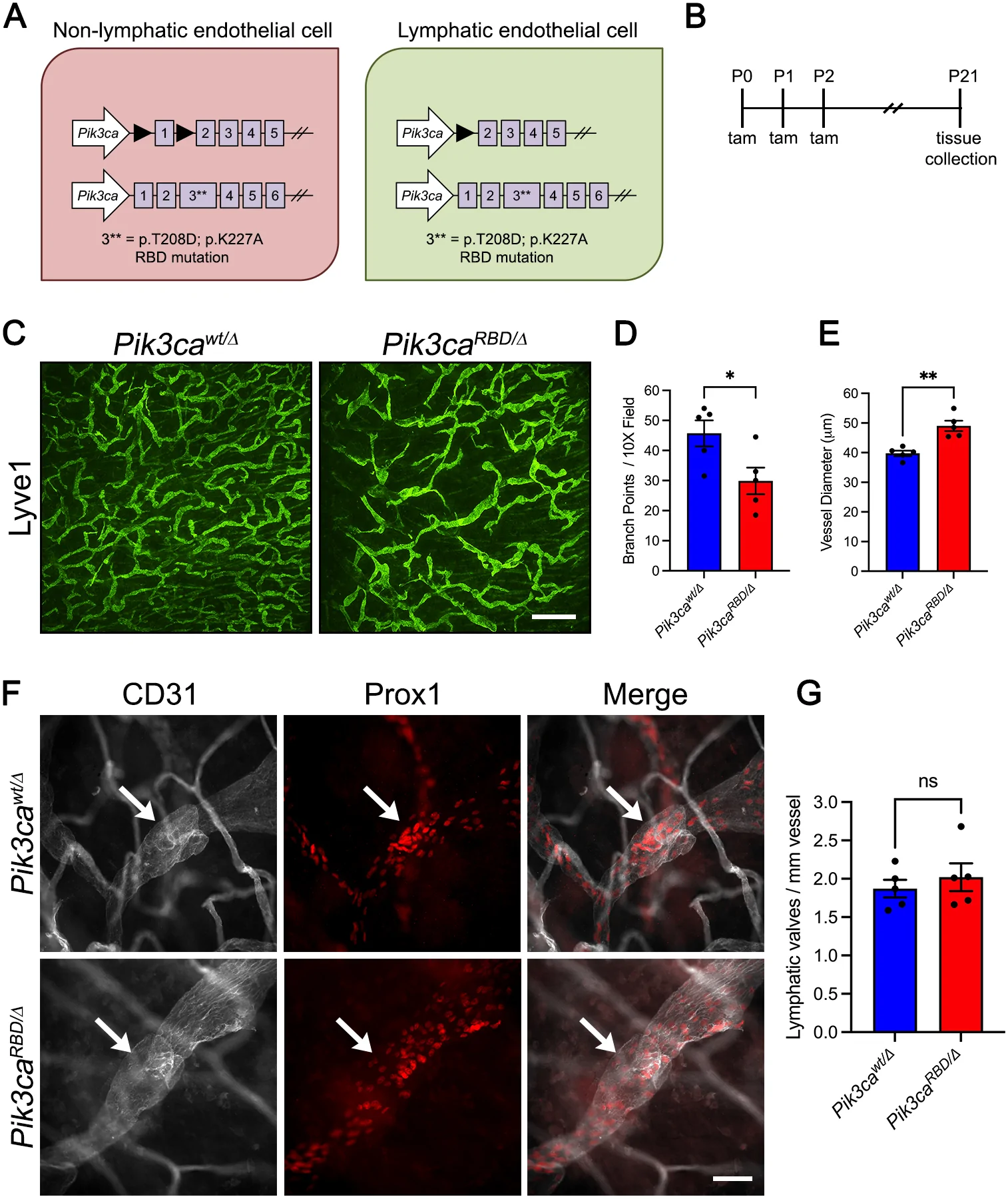
**Blocking the interaction between RAS and p110α in LECs impacts lymphatic branching but not lymphatic remodeling.** (A) Schematics of the *Pik3ca^flox^* and *Pik3ca^RBD^* alleles. Following tamoxifen administration, mice have either a single wild-type (*Pik3ca^wt/Δ^*) or mutant *Pik3ca* (*Pik3ca^RBD/Δ^*) allele in their LECs. (B) Schematic showing when mice received tamoxifen [50 μg, *per os* (p.o., by mouth)]. (C) Representative images of ear skin from 3-week-old mice immunostained for Lyve1. Scale bar: 500 μm. (D) Lymphatic branch points per 10× field in ears from *Flt4^wt/CreER^;Pik3ca^wt/Δ^* (45.70±4.31; *n*=5) and *Flt4^wt/CreER^;Pik3ca^RBD/Δ^* (29.90±4.44; *n*=5) mice. (E) Lymphatic diameters in ears from *Flt4^wt/CreER^;Pik3ca^wt/Δ^* (39.77±0.92; *n*=5) and *Flt4^wt/CreER^;Pik3ca^RBD/Δ^* (49.01±1.74; *n*=5) mice. (F) Representative images of ear skin whole mounts from 3-week-old mice immunostained for CD31 and Prox1. Arrows point to lymphatic valves. Scale bar: 50 μm. (G) Lymphatic valves per millimeter vessel in ears from *Flt4^wt/CreER^;Pik3ca^wt/Δ^* (1.87±0.12; *n*=5) and *Flt4^wt/CreER^;Pik3ca^RBD/Δ^* (2.02±0.18; *n*=5) mice. Data are presented as mean±s.e.m. *n*=number of mice. ns, not significant; *P<0.05; **P<0.01; unpaired two-tailed Student's *t*-tests (D,E,G).

To investigate the cell-autonomous role of RAS activation of p110α in lymphatic vessel development, we used the Cre-loxP system to knock out a floxed allele of *Pik3ca* in LECs (Fig. 3A). We bred *Pik3ca^wt/RBD^* mice with *Flt4^CreER/CreER^;Pik3ca^flox/flox^* mice to yield *Flt4^wt/CreER^;Pik3ca^wt/flox^* and *Flt4^wt/CreER^;Pik3ca^RBD/flox^* offspring. Following tamoxifen administration, mice have either a single wild-type (*Pik3ca^wt/Δ^*) or mutant (*Pik3ca^RBD/Δ^*) *Pik3ca* allele in their LECs (Fig. 3A). We fed newborn mice tamoxifen from P0 to P2 to induce recombination in *Flt4*-positive cells (Fig. 3B). We chose this tamoxifen regimen based on lineage-tracing experiments using *Flt4^wt/CreER^;mTmG* mice, which demonstrated that tamoxifen administration from P0 to P2 triggers widespread recombination in LECs (Fig. S3). Notably, nearly the entire lymphatic network of the ear (capillary, collector and valve LECs) was GFP positive, confirming robust recombination in LECs. Similar observations have also been reported for the skin, mesentery and diaphragms of *Flt4^wt/CreER^;mTmG* mice treated with tamoxifen on P0 and P2 (Mastrogiacomo et al., 2026). Although we observed robust recombination in LECs, we occasionally observed recombination in a small fraction of blood endothelial cells within the ear (Fig. S3). We collected ears from *Pik3ca^wt/Δ^* and *Pik3ca^RBD/Δ^* mice for whole-mount immunofluorescence staining approximately 3 weeks after feeding them tamoxifen (Fig. 3C). We found that *Pik3ca^RBD/Δ^* mice had significantly fewer lymphatic branch points than *Pik3ca^wt/Δ^* mice (Fig. 3D). Additionally, lymphatic vessel diameters were significantly greater in *Pik3ca^RBD/Δ^* mice than in *Pik3ca^wt/Δ^* mice (Fig. 3E). However, the number of lymphatic valves was not significantly different between *Pik3ca^RBD/Δ^* mice and *Pik3ca^wt/Δ^* mice (Fig. 3F,G). These results suggest that RAS activation of p110α in LECs is required for proper lymphatic vessel growth but not lymphatic vessel maturation.

### Blocking Kras^G12D^ activation of p110α decreases Kras^G12D^-induced enlargement of lymphatic vessels

**Fig. 4.**
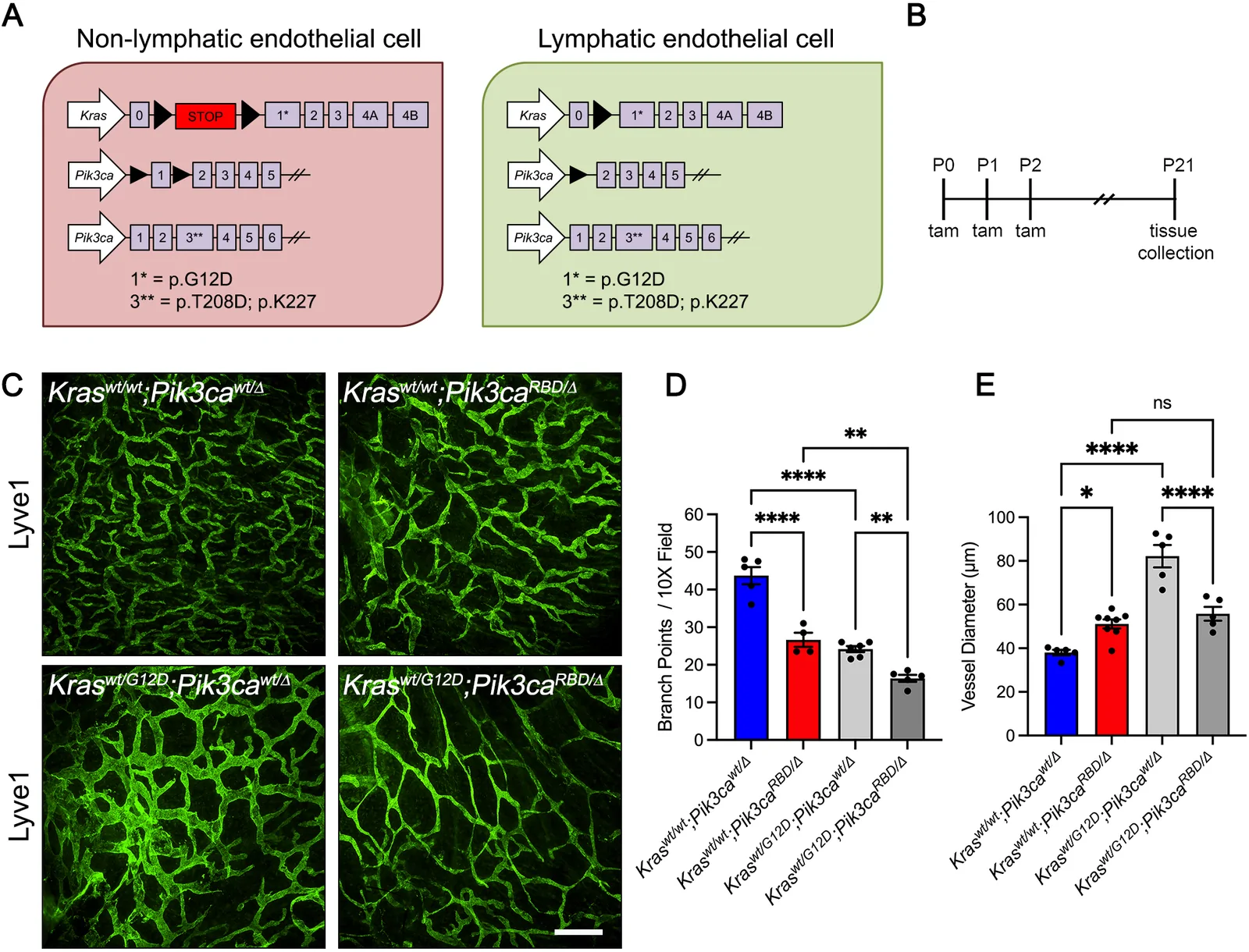
**Blocking Kras^G12D^ activation of p110α decreases Kras^G12D^-induced enlargement of lymphatic vessels.** (A) Schematics of the *Kras^G12D^*, *Pik3ca^flox^* and *Pik3ca^RBD^* alleles. Following tamoxifen administration, mice have either a single wild-type (*Pik3ca^wt/Δ^*) or mutant *Pik3ca* allele (*Pik3ca^RBD/Δ^*) in their LECs. They also express *Kras^G12D^* in their LECs. (B) Schematic showing when mice received tamoxifen (50 μg, p.o.). (C) Representative images of ear skin from 3-week-old mice immunostained for Lyve1. Scale bar: 500 μm. (D) Lymphatic branch points per 10× field in ears from *Flt4^wt/CreERT2^;Kras^wt/wt^;Pik3ca^wt/Δ^* (43.70±2.29; *n*=5), *Flt4^wt/CreERT2^;Kras^wt/wt^;Pik3ca^RBD/Δ^* (25.97±1.59; *n*=5), *Flt4^wt/CreERT2^;Kras^wt/G12D^;Pik3ca^wt/Δ^* (24.2±0.81; *n*=6) and *Flt4^wt/CreERT2^;Kras^wt/G12D^;Pik3ca^RBD/Δ^* (16.40±2.1; *n*=5) mice. (E) Lymphatic diameters in ears from *Flt4^wt/CreERT2^;Kras^wt/wt^;Pik3ca^wt/Δ^* (38.02±1.23; *n*=5), *Flt4^wt/CreERT2^;Kras^wt/wt^;Pik3ca^RBD/Δ^* (51.13±2.117; *n*=8), *Flt4^wt/CreERT2^;Kras^wt/G12D^;Pik3ca^wt/Δ^* (82.21±5.13; *n*=5), and *Flt4^wt/CreERT2^;Kras^wt/G12D^;Pik3ca^RBD/Δ^* (55.76±3.17; *n*=5) mice. Data are presented as mean±s.e.m. *n*=number of mice. ns, not significant; *P<0.05; **P<0.01; ****P<0.0001; one-way ANOVA with Tukey's multiple comparisons test (D,E).

It has previously been reported that the interaction between RAS and p110α is required for Kras^G12D^-induced tumor formation and maintenance (Castellano et al., 2013; Gupta et al., 2007). To determine whether blocking this interaction also impacts Kras^G12D^-induced lymphatic malformations, we bred the *Kras^LSL-G12D^* mutation into our mouse model (Fig. 4A). *Kras^LSL-G12D^* mice express an active form of *Kras* (Kras^G12D^) from the endogenous *Kras* locus following exposure to tamoxifen (Jackson et al., 2001). Again, we used the *Flt4^CreER^* line to induce recombination in LECs. We fed newborn mice tamoxifen from P0 to P2 to induce recombination in LECs (Fig. 4B). We then collected their ears for analysis when they were 3 weeks old (Fig. 4C). We found that lymphatic branching was lower in *Kras^wt/G12D^;Pik3ca^wt/Δ^* mice than in *Kras^wt/wt^;Pik3ca^wt/Δ^* mice (Fig. 4D). This phenotype was exacerbated by blocking Kras^G12D^ activation of p110α, as lymphatic branching was even lower in *Kras^wt/G12D^;Pik3ca^RBD/Δ^* mice compared to that in *Kras^wt/G12D^;Pik3ca^wt/Δ^* mice (Fig. 4D).

Next, we examined the role of Kras^G12D^ activation of p110α in regulating the size of lymphatic vessels. We found that lymphatic diameters were significantly greater in *Kras^wt/G12D^;Pik3ca^wt/Δ^* mice than in *Kras^wt/wt^;Pik3ca^wt/Δ^* mice (Fig. 4E). Interestingly, lymphatic vessel diameters were significantly smaller in *Kras^wt/G12D^;Pik3ca^RBD/Δ^* mice compared to those in *Kras^wt/G12D^;Pik3ca^wt/Δ^* mice (Fig. 4E).

**Fig. 5.**
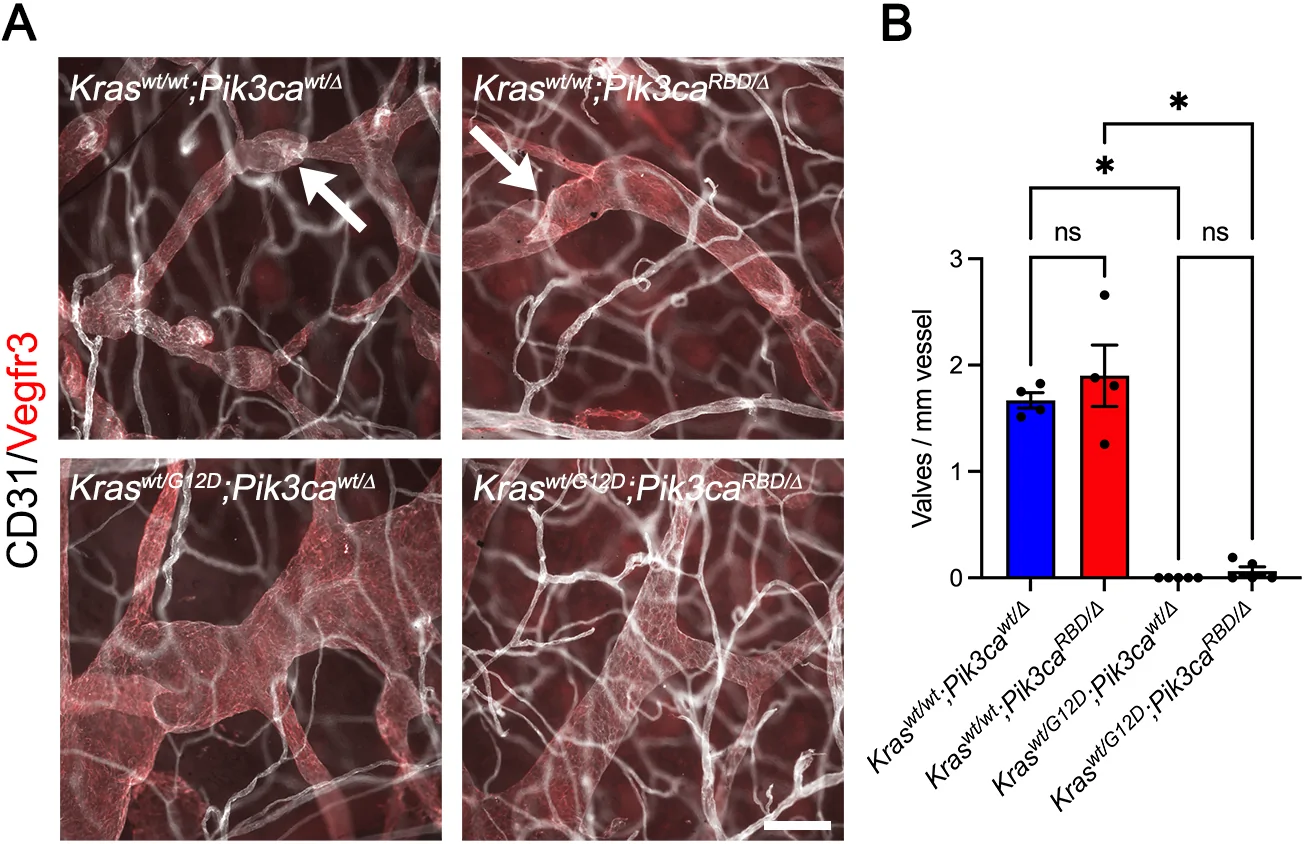
**Blocking Kras^G12D^ activation of p110α does not impact the effects of Kras^G12D^ on lymphatic valves.** (A) Representative images of ear skin from 3-week-old mice immunostained for CD31 and Vegfr3. Arrows point to valves. Scale bar: 100 μm. (B) Lymphatic valves per millimeter vessel in ears from *Flt4^wt/CreERT2^;Kras^wt/wt^;Pik3ca^wt/Δ^* (1.67±0.07; *n*=4), *Flt4^wt/CreERT2^;Kras^wt/wt^;Pik3ca^RBD/Δ^* (1.90±0.29; *n*=4), *Flt4^wt/CreERT2^;Kras^wt/G12D^;Pik3ca^wt/Δ^* (0.00±0.00; *n*=5) and *Flt4^wt/CreERT2^;Kras^wt/G12D^;Pik3ca^RBD/Δ^* (0.07±0.04; *n*=5) mice. Data are presented as mean±s.e.m. *n*=number of mice. ns, not significant; *P<0.05; Kruskal–Wallis nonparametric test with Dunn's multiple comparisons test.

We previously reported that Kras^G12D^ expression in LECs impairs the formation of lymphatic valves (Fernandes et al., 2025, 2023; Homayun-Sepehr et al., 2021; Mastrogiacomo et al., 2026). To determine whether blocking Kras^G12D^ activation of p110α would allow lymphatic valves to form, we analyzed valves in ear skin samples from 3-week-old mice (Fig. 5A). We found that *Kras^wt/G12D^;Pik3ca^wt/Δ^* mice had significantly fewer lymphatic valves than *Kras^wt/wt^;Pik3ca^wt/Δ^* mice (Fig. 5B). Importantly, we found that the number of lymphatic valves was also dramatically reduced in *Kras^wt/G12D^;Pik3ca^RBD/Δ^* mice (Fig. 5B). These findings indicate that blocking Kras^G12D^-mediated activation of p110α does not restore valve formation in Kras^G12D^-mutant mice.

To determine whether lymphatic vessel abnormalities in *Kras^wt/G12D^;Pik3ca^RBD/Δ^* mice were associated with altered LEC proliferation, we stained ear skin from control and mutant mice with antibodies against Ki-67 (encoded by *Mki67*) and Prox1. Mice received tamoxifen from P0 to P2, and ears were collected at P15 to examine LEC proliferation during active lymphatic vessel development. We found that the percentage of proliferating LECs was greater in *Kras^wt/G12D^;Pik3ca^wt/Δ^* mice compared to that in *Kras^wt/wt^;Pik3ca^wt/Δ^* mice (Fig. S4). Additionally, we found that *Kras^wt/G12D^;Pik3ca^RBD/Δ^* mice had significantly fewer proliferating LECs than *Kras^wt/G12D^;Pik3ca^wt/Δ^* mice (Fig. S4). These results suggest that blocking RAS activation of p110α reduces Kras^G12D^-induced LEC proliferation.

### Deletion of p110α has a similar effect on Kras^G12D^-induced lymphatic malformations as blocking RAS activation of p110α

**Fig. 6.**
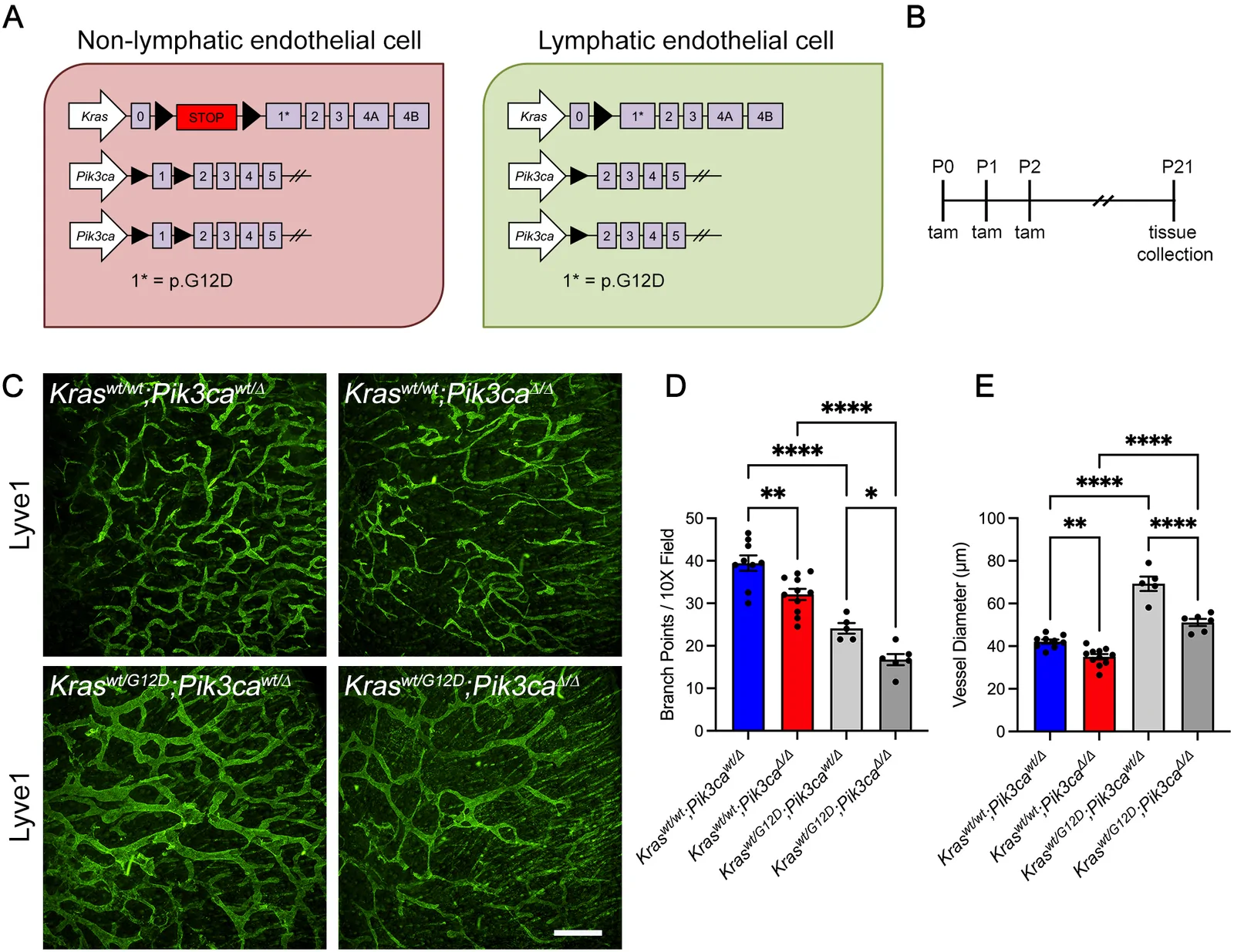
**Deleting p110α decreases Kras^G12D^-induced enlargement of lymphatic vessels.** (A) Schematics of the *Kras^G12D^* and *Pik3ca^flox^* alleles. Following tamoxifen administration, mice either lack *Pik3ca* (Pik3ca*^Δ/Δ^*) or have a single copy of *Pik3ca* (*Pik3ca^wt/Δ^*) in their LECs. They also express *Kras^G12D^* in their LECs. (B) Schematic showing when mice received tamoxifen (50 μg, p.o.). (C) Representative images of ear skin from 3-week-old mice immunostained for Lyve1. Scale bar: 500 μm. (D) Lymphatic branch points per 10× field in ears from *Flt4^wt/CreERT2^;Kras^wt/wt^;Pik3ca^wt/Δ^* (39.44±1.81; *n*=9), *Flt4^wt/CreERT2^;Kras^wt/wt^;Pik3ca^Δ/Δ^* (32.09±1.31; *n*=11), *Flt4^wt/CreERT2^;Kras^wt/G12D^;Pik3ca^wt/Δ^* (24.10±1.24; *n*=5) and *Flt4^wt/CreERT2^;Kras^wt/G12D^;Pik3ca^Δ/Δ^* (16.75±1.32; *n*=6) mice. (E) Lymphatic diameters in ears from *Flt4^wt/CreERT2^;Kras^wt/wt^;Pik3ca^wt/Δ^* (42.16±1.00; *n*=9), *Flt4^wt/CreERT2^;Kras^wt/wt^;Pik3ca^Δ/Δ^* (35.04±1.24; *n*=11), *Flt4^wt/CreERT2^;Kras^wt/G12D^;Pik3ca^wt/Δ^* (69.27±3.36; *n*=5) and *Flt4^wt/CreERT2^;Kras^wt/G12D^;Pik3ca^Δ/Δ^* (51.14±1.67; *n*=6) mice. Data are presented as mean±s.e.m. *n*=number of mice. *P<0.05; **P<0.01; ****P<0.0001; one-way ANOVA with Tukey's multiple comparisons test (D,E).

**Fig. 7.**
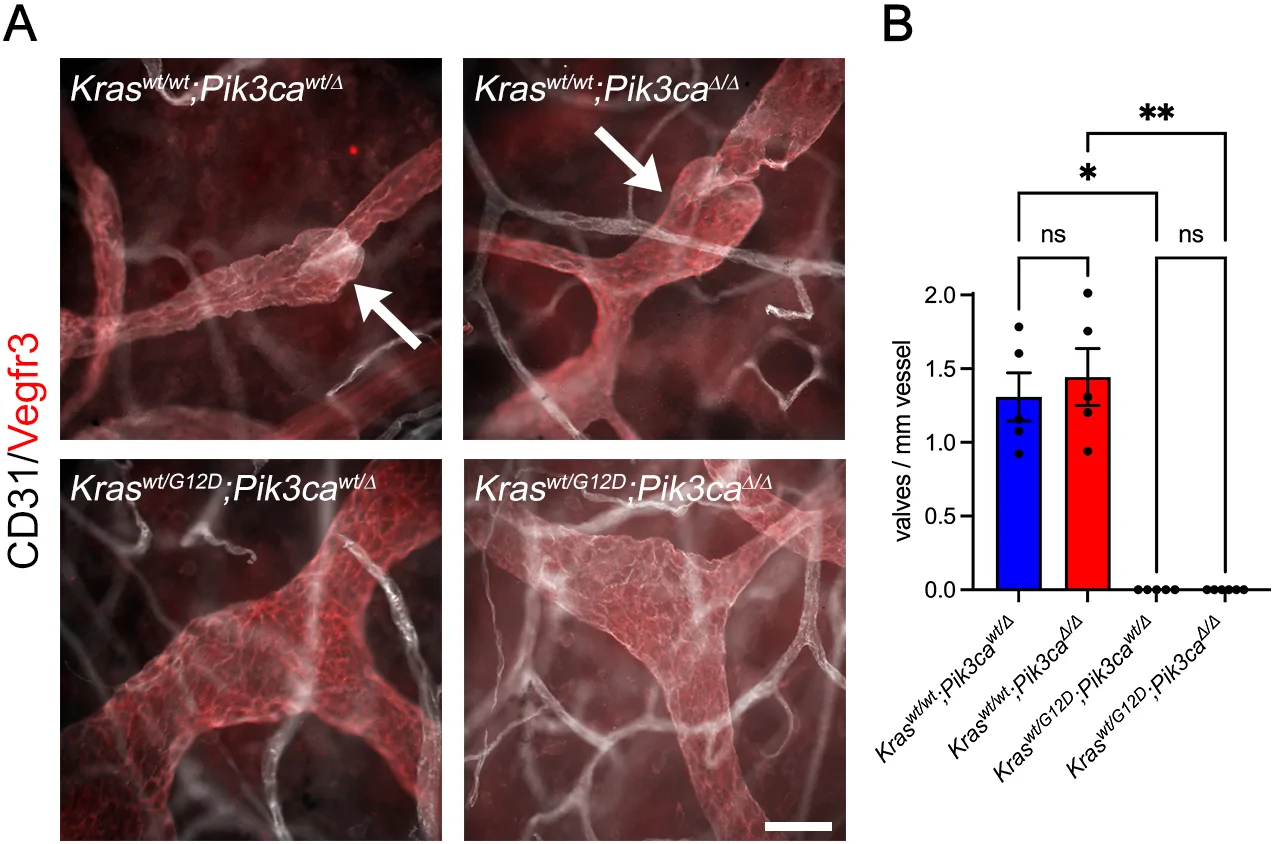
**Deleting p110α does not impact the effects of Kras^G12D^ on lymphatic valves.** (A) Representative images of ear skin from 3-week-old mice immunostained for CD31 and Vegfr3. Arrows point to valves. Scale bar: 100 μm. (B) Lymphatic valves per millimeter vessel in ears from *Flt4^wt/CreERT2^;Kras^wt/wt^;Pik3ca^wt/Δ^* (1.31±0.16; *n*=5), *Flt4^wt/CreERT2^;Kras^wt/wt^;Pik3ca^Δ/Δ^* (1.44±0.19; *n*=5), *Flt4^wt/CreERT2^;Kras^wt/G12D^;Pik3ca^wt/Δ^* (0.0±0.0; *n*=5) and *Flt4^wt/CreERT2^;Kras^wt/G12D^;Pik3ca^Δ/Δ^* (0.0±0.0; *n*=6) mice. Data are presented as mean±s.e.m. *n*=number of mice. ns, not significant; *P<0.05; **P<0.01; Kruskal–Wallis nonparametric test with Dunn's multiple comparisons test.

To determine whether deleting p110α has a different effect on Kras^G12D^-induced lymphatic malformations compared to blocking Kras^G12D^-mediated activation of p110α, we generated *Kras^wt/G12D^;Pik3ca^Δ/Δ^* mice (Fig. 6A). The *Flt4^CreER^* line was used to induce recombination in LECs. We treated control and mutant mice with tamoxifen from P0 to P2 and then collected ears for whole-mount immunostaining on P21 (Fig. 6B). We found that the number of lymphatic branch points and the diameter of lymphatic vessels were significantly lower in *Kras^wt/G12D^;Pik3ca^Δ/Δ^* mice compared to those in *Kras^wt/G12D^;Pik3ca^wt/Δ^* mice (Fig. 6C-E). Additionally, we found that the number of lymphatic valves was reduced in *Kras^wt/G12D^;Pik3ca^Δ/Δ^* and *Kras^wt/G12D^;Pik3ca^wt/Δ^* mice (Fig. 7A,B). These results indicate that deleting p110α has a similar effect on Kras^G12D^-induced lymphatic malformations as blocking RAS activation of p110α.

## DISCUSSION

**Fig. 8.**
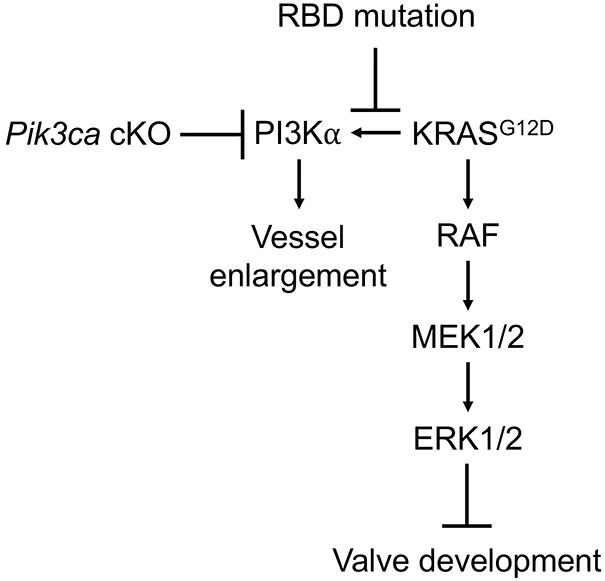
**Working model for the role of RAS–PI3Kα signaling in Kras^G12D^-induced complex lymphatic anomalies.** Our results indicate that Kras^G12D^ induces distinct disease phenotypes by signaling through separate pathways. Specifically, Kras^G12D^–PI3Kα signaling leads to the pathological enlargement of lymphatic vessels, whereas Kras^G12D^–MAPK signaling impairs the formation of lymphatic valves. cKO, conditional knockout.

Active KRAS promotes cell signaling by interacting with several downstream effector proteins, including PI3Kα. However, the role KRAS–PI3Kα signaling serves in the pathogenesis of CLAs was incompletely understood. In this study, we found that blocking the interaction between p110α and Kras^G12D^ decreased the pathological enlargement of lymphatic vessels in Kras^G12D^-mutant mice. However, it did not prevent the loss of lymphatic valves in Kras^G12D^-mutant mice. Our results shed light on the molecular mechanisms by which Kras^G12D^ causes CLAs and suggest that Kras^G12D^–PI3Kα signaling regulates lymphatic vessel size, whereas Kras^G12D^–MAPK signaling controls the formation of lymphatic valves (Fig. 8).

To characterize class 1 PI3K gene expression in LECs, we analyzed scRNA-seq data from murine LECs and bulk RNA-seq data from human LECs. This approach revealed that *Pik3ca/PIK3CA* and *Pik3r1/PIK3R1* are the most abundantly expressed class 1 PI3K transcripts in both mouse and human LECs. It is important to note, however, that transcript levels may not accurately reflect protein abundance or signaling activity. Furthermore, our analysis of human LECs was limited to cells cultured *ex vivo*. Future studies utilizing scRNA-seq of freshly isolated human lymphatic vessels could provide a more accurate assessment of class 1 PI3K transcript abundance *in vivo*.

The *Pik3ca^RBD^* mice used in our study harbor two germline missense mutations (p.T208D and p.K227A) in the RAS-binding domain of p110α (Gupta et al., 2007). These mutations block the interaction between p110α and RAS proteins but do not affect the kinase activity of p110α (Gupta et al., 2007). A group previously reported that *Pik3ca^RBD/RBD^* mice exhibit lymphatic defects, such as chylous ascites and lymphatic hypoplasia, suggesting that RAS–PI3K signaling is crucial for lymphatic vessel development (Gupta et al., 2007). However, because RAS–PI3Kα signaling is impaired in all cells in *Pik3ca^RBD/RBD^* mice, it was unclear whether the lymphatic defects were due to cell-autonomous or non-cell-autonomous effects. By combining the Cre-loxP system with the *Pik3ca^RBD^* allele, we were able to analyze mice that lacked RAS-mediated activation of PI3Kα specifically in LECs. Importantly, our experiments demonstrate that cell-autonomous RAS–PI3Kα signaling in LECs is required for the sprouting but not the maturation of lymphatic vessels.

Lymphatic vessel branching is a complex process regulated by VEGFC–VEGFR3 (also known as FLT4) signaling (Ucar et al., 2023). In the mouse ear, lymphatic vessels first form a rough network and then actively send side branches into poorly covered areas (Ucar et al., 2023). The side-branching of lymphatic vessels results in the efficient coverage of tissues by lymphatic vessels and is controlled by VEGFC–VEGFR3 signaling (Ucar et al., 2023). Similar to *Vegfc^wt/-^* mice, *Pik3ca^Δ/RBD^* mice also exhibit a reduction in lymphatic branch points. Recent studies have shown that PI3Kα signaling promotes the cell surface expression of VEGFR3 (Korhonen et al., 2022). Consequently, disruption of RAS-mediated activation of p110α may impair lymphatic vessel branching and LEC proliferation by limiting VEGFR3 availability at the cell surface (Korhonen et al., 2022). Interestingly, immunostaining for VEGFR3 revealed that Kras^G12D^ expression by LECs can increase or decrease VEGFR3 intensity in a tissue-specific manner (Mastrogiacomo et al., 2026). Future experiments examining VEGFR3 localization will help clarify the specific role of RAS-mediated activation of p110α in regulating cell surface VEGFR3 levels.

Our results provide additional evidence that RAS–MAPK signaling plays a critical role in the development of lymphatic valves (Fernandes et al., 2023; Homayun-Sepehr et al., 2021; Mastrogiacomo et al., 2026). They also complement other work demonstrating that loss of *Rasa1*, a negative regulator of RAS signaling, increases MAPK signaling and impairs lymphatic valve development in mice (Chen et al., 2020, 2024; Lapinski et al., 2017). The importance of RASA1 in regulating lymphatic valve formation has also been observed in other species. Zebrafish have two *rasa1* genes (*rasa1a* and *rasa1b*), and *rasa1a;rasa1b* double-mutant fish exhibit defects in the formation of lymphovenous valves and facial lymphatic valves (Meng et al., 2023). To dynamically monitor MAPK activity during lymphatic valve development in zebrafish, a transgenic ERK kinase translocation reporter line was created (Meng et al., 2023). The localization of this unique, fluorescent biosensor is controlled by ERK1/2 activity. The biosensor localizes to the cytoplasm when ERK1/2 is active and to the nucleus when ERK1/2 is inactive (Meng et al., 2023). Using this biosensor, investigators found a regional and temporal decrease in ERK1/2 activity during lymphatic valve development in zebrafish (Meng et al., 2023). Because mutations that cause hyperactive ERK1/2 signaling impair lymphatic valve development in mice, it seems highly likely that ERK1/2 is also regionally and temporally decreased in valve-forming regions in mammals. In the future, ERK biosensors or immunostaining for phospho-ERK1/2 and lymphatic valve markers could reveal whether ERK1/2 activity is decreased in valve-forming regions in mammals as it is in zebrafish.

In addition to KRAS, activating variants in HRAS and NRAS can cause CLAs (Barclay et al., 2019; Li et al., 2023; Manevitz-Mendelson et al., 2018; Ozeki et al., 2019). Furthermore, overexpression of HRAS or expression of an active form of NRAS in LECs induces lymphatic malformations in mice (Ichise et al., 2010; McDaniel et al., 2025; Nozawa et al., 2024). KRAS, HRAS, and NRAS are highly conserved proteins, with their major differences occurring in the last 20 C-terminal amino acids, known as the hypervariable region (Ahearn et al., 2012). Despite this substantial sequence similarity, RAS proteins exhibit striking differences in signaling. KRAS, NRAS and HRAS differ in their ability to activate RAF1, with KRAS exhibiting the strongest ability and HRAS displaying the weakest ability (Voice et al., 1999). KRAS and HRAS have also been reported to differ in their ability to activate PI3K, with HRAS being a more potent activator of PI3K than KRAS in an *in vitro* assay (Yan et al., 1998). However, a limitation of that study was that it did not resolve isoform-specific PI3K behavior (e.g. PI3Kα, PI3Kβ or PI3Kδ). More recent work has shown that KRAS and NRAS bind PI3Kα with a greater affinity than HRAS, whereas RRAS2 and MRAS have an even higher affinity than any of the classical RAS proteins (Czyzyk et al., 2025). In the future, our approach with *Pik3ca^RBD^* mice could be used to determine whether blocking NRAS- or HRAS-mediated PI3Kα activation has a more pronounced effect on lymphatic malformations than KRAS.

PI3Kα inhibitors are gaining popularity for the treatment of CLAs, particularly for patients with an activating *PIK3CA* (p110α) variant (Grenier et al., 2023; Li et al., 2023). Alpelisib is a p110α-selective inhibitor that binds to the ATP-binding site of p110α. Response to alpelisib varies among patients with CLA with a *PIK3CA* variant. Some show a significant improvement in disease, whereas others experience only a modest improvement or disease progression (Grenier et al., 2023; Li et al., 2023). Inavolisib is another FDA-approved p110α-selective inhibitor that, unlike alpelisib, also promotes the degradation of mutant p110α (Song et al., 2025). Although its use in CLAs has not yet been reported, it represents a compelling candidate for future therapeutic exploration. The effect of PI3K inhibitors on CLAs caused by RAS variants has not been widely investigated. Our work suggests that blocking RAS activation of PI3Kα alone may not be an effective strategy for treating CLAs caused by *KRAS* variants because it did not lead to the formation of lymphatic valves. However, combination therapy with PI3Kα and MEK1/2 inhibitors may be a more efficacious approach than monotherapy for patients with a RAS variant. BBO-10203 is a new PI3Kα inhibitor, termed a RAS::PI3K breaker, which covalently binds near the RAS-binding domain of p110α and blocks the interaction between RAS and p110α (Simanshu et al., 2025). Thus, BBO-10203 behaves similarly to the *Pik3ca^RBD^* mutation. Importantly, BBO-10203 does not cause hyperglycemia like other PI3K inhibitors (Simanshu et al., 2025). In the future, BBO-10203 may be a stronger candidate than other PI3K inhibitors for combination therapy with MEK1/2 inhibitors for the treatment of RAS-driven CLAs. Furthermore, it may also be useful for PIK3CA-variant CLAs as it has been reported to reduce AKT phosphorylation in PIK3CA^H1047R^-, PIK3CA^E542K^- and PIK3CA^E545K^-mutant cell lines (Simanshu et al., 2025).

Recent cryo-electron microscopy experiments have provided insights into the mechanism of PI3Kα activation (Torosyan et al., 2026). The authors propose that PI3Kα activation occurs in stages, ultimately leading to the dimerization of KRAS–PI3Kα complexes to promote downstream signaling (Torosyan et al., 2026). One of the early steps in this model is the recruitment of PI3Kα to the membrane by KRAS. Thus, the formation of KRAS–PI3Kα complex dimers could be impaired in the *Pik3ca^RBD^* mice used in our study.

In our study, we used a genetic approach to investigate the cell-autonomous role of RAS–PI3Kα signaling in lymphatic vessel development. One limitation of this approach is that LECs expressing the RAS-binding-deficient *Pik3ca* allele are effectively hemizygous for *Pik3ca* following deletion of the wild-type allele. Therefore, we cannot formally exclude a contribution of reduced *Pik3ca* gene dosage to the observed phenotype. However, the lymphatic vasculature appears overtly normal in *Pik3ca^wt/Δ^* mice, suggesting that *Pik3ca* hemizygosity in LECs is insufficient to markedly impair lymphatic vessel development. A second limitation is that *Pik3ca^flox/RBD^* mice are heterozygous for the *Pik3ca^RBD^* allele in all cell types prior to Cre-mediated recombination. Importantly, *Pik3ca^wt/RBD^* embryos and weanlings are grossly indistinguishable from controls and exhibit normal lymphatic development. Although we observed a modest reduction in vessel diameter in *Pik3ca^wt/RBD^* weanlings, this difference was minor relative to the defects observed following expression of the *Pik3ca^RBD^* allele in the absence of the wild-type allele, suggesting that systemic heterozygosity for the *Pik3ca^RBD^* allele is insufficient to substantially impair lymphatic development. Thus, although subtle contributions from non-LEC populations cannot be completely excluded, our findings support a predominantly cell-autonomous requirement for RAS-mediated PI3Kα signaling in LECs.

Another consideration is that our studies utilized widespread induction of *Kras^G12D^* expression in LECs. We chose this approach because it provides a robust system for investigating the mechanisms by which Kras^G12D^ and its downstream signaling pathways contribute to defects in vessel morphogenesis. However, the fraction of mutant LECs present within human lymphatic malformations remains incompletely defined. Available sequencing data suggest that some patients harbor low-level mosaicism for CLA-causing variants (Li et al., 2023). Therefore, the high recombination efficiency achieved in our model may not fully recapitulate disease states characterized by lower variant allele frequencies. Future studies using lower tamoxifen doses may help define how mutant cell burden influences disease pathogenesis. Lastly, although our Cre line and tamoxifen strategy result in occasional recombination in a small fraction of blood endothelial cells in the ear, recombination is widespread throughout the lymphatic vasculature. We did not observe overt vascular abnormalities in the ear vasculature, suggesting that the limited recombination in blood endothelial cells is unlikely to be a major driver of the lymphatic phenotypes in mutant mice.

In conclusion, our results indicate that Kras^G12D^ induces distinct disease phenotypes by signaling through separate pathways. Specifically, Kras^G12D^–PI3Kα signaling leads to the pathological enlargement of lymphatic vessels, whereas Kras^G12D^–MAPK signaling impairs the formation of lymphatic valves. Together, our results shed light on the mechanisms by which KRAS variants cause CLAs and have implications for the treatment of these life-threatening diseases.

## MATERIALS AND METHODS

### Sex as a biological variable

Both male and female mice were examined in our study, and we obtained similar results for both sexes.

### Description of mouse strains

The animal experiments described in this study were carried out in accordance with an animal protocol approved by the Institutional Animal Care and Use Committee of University of Texas Southwestern Medical Center. Mice were maintained in ventilated microisolator cages and were fed a standard diet. Mice were provided igloos and nestlets as enrichment items. Male and female mice were used in experiments. *Flt4^CreERT2^*, *Pik3ca^RBD^*, *Pik3ca^flox^*, *mT/mG* and *Kras^LSL-G12D^* mice have been described previously (Gupta et al., 2007; Jackson et al., 2001; Martinez-Corral et al., 2016; Muzumdar et al., 2007; Zhao et al., 2006).

### Tamoxifen treatment

We dissolved tamoxifen (25 mg; MilliporeSigma, T5648) in a mixture of ethanol (100 μl; MilliporeSigma, E7023) and peanut oil (900 μl; MilliporeSigma, P2144). Newborn mice were fed 2 μl of tamoxifen on P0, P1 and P2 using a P20 pipette.

### Analysis of scRNA-seq data

scRNA-seq datasets spanning multiple developmental timepoints from E14.5, E16.5, E18.5 to P0 were obtained from a publicly available repository (GSE213744; Fu et al., 2023). The P21 dataset used in this study was previously generated in-house using the 10× Genomics Chromium platform (Fernandes et al., 2025).

Using R (https://www.R-project.org/), we performed the analysis with the Seurat package (v4.4.0) (Hao et al., 2021). Seurat objects were created for each dataset using raw count matrices, which were obtained by reading the datasets from raw feature-barcode matrices or HDF5 files, respectively. Genes detected in fewer than three cells were excluded while creating Seurat objects. The datasets were then merged into a single Seurat object using the merge function. Cells were filtered based on standard quality control (QC) metrics. Cells with more than 500 detected genes and less than 5% mitochondrial transcript content were retained for downstream analysis. QC distributions were visualized using violin and feature scatter plots to confirm filtering thresholds.

Following QC, datasets were batch corrected and integrated using the data integration workflow in Seurat. The combined object was split by sample identity, and each dataset was independently normalized using the log-normalization method (‘NormalizeData’). Highly variable genes were identified within each dataset using the ‘FindVariableFeautures’ function (selection.method=“vst”, nfeatures=2000). Batch correction across developmental stages was performed using the anchor-based canonical correlation analysis integration workflow in Seurat. To find integration features shared across datasets, the ‘SelectIntegrationFeatures’ function was employed. Pairwise integration anchors were computed using the ‘FindIntegrationAnchors’ function, and datasets were integrated using the ‘IntegrateData’ function.

Integrated expression values were scaled using ‘ScaleData’ function. Principal component analysis was performed with ‘RunPCA’. First, 30 principal components were computed. After inspection of an elbow plot, the first 25 components were used for neighbor graph construction and visualization. The first 25 principal components were used to construct uniform manifold approximation and projection embeddings with the ‘RunUMAP’ function. The ‘FindNeighbors’ function was used to construct nearest-neighbor graphs, and unsupervised clustering was performed with ‘FindClusters’, which employs the Louvain algorithm. Resolution parameters were optimized empirically at each analysis stage.

Clusters lacking uniform expression (i.e. low percentage expression) of the LEC identity marker *Prox1* or showing low RNA complexity suggestive of damaged or senescent cells were iteratively removed to ensure consistency with LECs for further analysis. The remaining cells were re-normalized and re-integrated following each filtering step to minimize batch effects introduced by subsetting. Additionally, we subset cells with expression levels of *Vegfr3* above zero (*Flt4*>0) and levels of *Vegfr1* equal to zero (*Flt1*=0). These filtering steps were included to guarantee the best quality and purity of LECs for downstream analysis. Dimensionality reduction and clustering were repeated on the refined dataset.

Clusters were annotated based on expression of established lymphatic endothelial subtype markers (Fernandes et al., 2025; Gonzalez-Loyola et al., 2021; Petkova et al., 2023). Related clusters were grouped into broader LEC subtypes reflecting Ptx3 capillary, capillary, collecting, valve, proliferating and mixed LEC clusters. Developmental stage metadata were used to order samples chronologically. The final refined, integrated and annotated Seurat object was saved as an RDS file for downstream analyses. The RDS file was used to generate the dot plots for this study.

### Antibodies

The following primary antibodies were used for immunofluorescence staining: goat anti-Lyve1 (R&D Systems, AF2125; 1:500; RRID:AB_2297188), rabbit anti-Lyve1 (Cell Signaling, 67538; 1:1000; RRID:AB_3750510), goat anti-Vegfr3 (R&D Systems, AF743; 1:250; RRID:AB_355563), goat anti-Prox1 (R&D Systems, AF2727; 1:500; RRID:AB_2170716), rat anti-CD31 (BD Biosciences, 553370; 1:1000; RRID:AB_394816), rabbit anti-Ki-67 (Abcam, ab15580; 1:1000; RRID:AB_443209) and rabbit anti-neuropilin-2 (Cell Signaling, 3366; 1:500; RRID:AB_2155250). All secondary antibodies were used at 1:500. The following secondary antibodies were used for immunofluorescence staining: Cy3-conjugated donkey anti-rabbit (Jackson Immunoresearch, 711-165-152; RRID:AB_2307443), Cy3-conjugated donkey anti-goat (Jackson Immunoresearch, 705-165-003; RRID:AB_2340411), FITC-conjugated donkey anti-rabbit (Jackson Immunoresearch, 711-095-152; RRID:AB_2315776), FITC-conjugated donkey anti-chicken (Jackson Immunoresearch, 703-095-155; RRID:AB_2340356), Cy5-conjugated donkey anti-goat (Jackson Immunoresearch, 705-175-147; RRID:AB_2340415) and Cy5-conjugated donkey anti-rat (Jackson Immunoresearch, 712-095-150; RRID:AB_2340651).

### Whole-mount immunofluorescence staining

Whole-mount immunofluorescence staining of back and ear skin was performed as described previously (Fernandes et al., 2023). Briefly, skin samples were collected and fixed overnight in 1% paraformaldehyde (ear skin) or 4% paraformaldehyde. The fixed samples were washed with PBS and then permeabilized using PBS containing 0.3% Triton X-100 (PBST). Following this, the samples were blocked overnight with PBST supplemented with 3% donkey serum. The next day, they were incubated overnight with primary antibodies diluted in PBST. After thorough washing (three rounds of 40 min each) with PBST, the samples were treated overnight with secondary antibodies diluted in PBST. After another series of washes with PBST (three rounds of 40 min each), the samples were transferred to glass slides, and coverslips were mounted using ProLong Gold (Invitrogen, 36934).

### Immunofluorescence staining of tissue sections

Slides were heated in a 60°C oven, deparaffinized with xylene, and rehydrated through a descending ethanol series (100%, 95%, 70%, 50%, 0%). Non-specific binding was blocked by incubating slides in TBS containing 0.2% Tween 20 (TBST) containing 3% donkey serum for 30 min. Slides were then incubated overnight with primary antibodies diluted in TBST containing 5% BSA. Slides were washed with TBST and then incubated with secondary antibodies diluted in TBST containing 5% BSA. Following washes with TBST, coverslips were mounted with ProLong Gold containing DAPI (Invitrogen, 36935).

### Image acquisition

Samples were analyzed using a Nikon Eclipse E600 epifluorescence microscope equipped with filter sets for FITC, Cy3, Cy5 and DAPI, and an X-Cite 120 fluorescence illumination system. Images were captured using a Nikon DS-Qi2 camera mounted on the microscope. Image acquisition and analysis were performed using NIS Elements software (version 5.30.02; Nikon). All images within each experiment were acquired using similar settings. Post-acquisition processing was limited to uniform adjustments in brightness and contrast applied across all conditions.

### Analysis of whole-mount skin preparations

#### Analysis of embryonic back skin

We captured one image at 4× magnification per embryo for midline gap and branch point measurements. The images were taken towards the center of the back where the midline is uniformly spaced. This allows for more consistent midline gap and branch point measurements. We then manually counted the lymphatic branch points and traced the midline gap. Vessel diameters were measured from one image at 4× magnification per mouse in most cases. For mice with two images, measurements were averaged to generate a single value for each mouse. To measure vessel diameters, we placed a 100 μm×100 μm grid over the images and measured the diameter of vessels located on intersecting grid lines. We used NIS Elements software to make all measurements. Image analysis was performed in a masked manner.

#### Analysis of ear skin

To assess branch points, we manually counted the number of lymphatic branch points in distal regions of the ear using 4× (one image per mouse) or 10× (two images per mouse) image fields, as indicated in the figure legends. To measure vessel diameters, we overlaid a 200 μm×200 μm grid on 4× images (one image per mouse) or a 100 μm×100 μm grid on 10× images (two images per mouse) and measured the diameters of lymphatic vessels located at grid intersections. To quantify lymphatic valves, we measured the total length of the lymphatic network and manually counted the number of lymphatic valves in proximal ear regions using 10× (two images per mouse) or 20× (three to four images per mouse) images. For analyses in which multiple images were acquired from a mouse, measurements were averaged to generate a single value per mouse for statistical analysis. All measurements were performed using NIS-Elements software. Image analysis was performed in a masked manner.

### Analysis of LEC proliferation

Prox1-positive LECs were quantified from three representative 20× fields per ear. Within the same fields, LECs co-expressing Prox1 and Ki-67 were identified and counted. The percentage of Ki-67-positive LECs was then calculated for each ear by dividing the number of Prox1 and Ki-67 double-positive LECs by the total number of Prox1-positive LECs and multiplying by 100. Image analysis was performed in a masked manner.

### Statistical analysis

GraphPad Prism (Version 9.5.1) was used for statistical analysis. One-way ANOVA was used to evaluate differences between three or more groups. The Shapiro–Wilk test was used to determine normality. When the assumptions of normality were not met, we used the Kruskal–Wallis nonparametric test for three or more groups, followed by Dunn's multiple comparisons test. When the assumptions of normality were met, we used one-way ANOVA with Tukey's multiple comparisons test. Unpaired two-tailed Student's *t*-tests were performed when comparing differences between two groups to test means for significance. Chi-square (χ^2^) goodness-of-fit test was performed to determine if observed genotype frequencies deviated from expected Mendelian ratios. All results are expressed as mean±s.e.m. The number of mice in each group is indicated in the figure legends (*n*=number of mice). A *P*-value of less than 0.05 was considered significant.

## Supplementary Material

10.1242/dmm.052932_sup1 Supplementary information
