# Supplementary material for: RAS–PI3Kα signaling regulates KrasG12D-induced lymphangiogenesis in complex lymphatic anomalies
Source: Dis Model Mech. 2026 Aug 27;19(8):dmm052932. doi: 10.1242/dmm.052932 (PMC13580581; doi:10.1242/dmm.052932)
Supplement: Supplementary file 1 [file dmm-19-052932-s1.pdf]

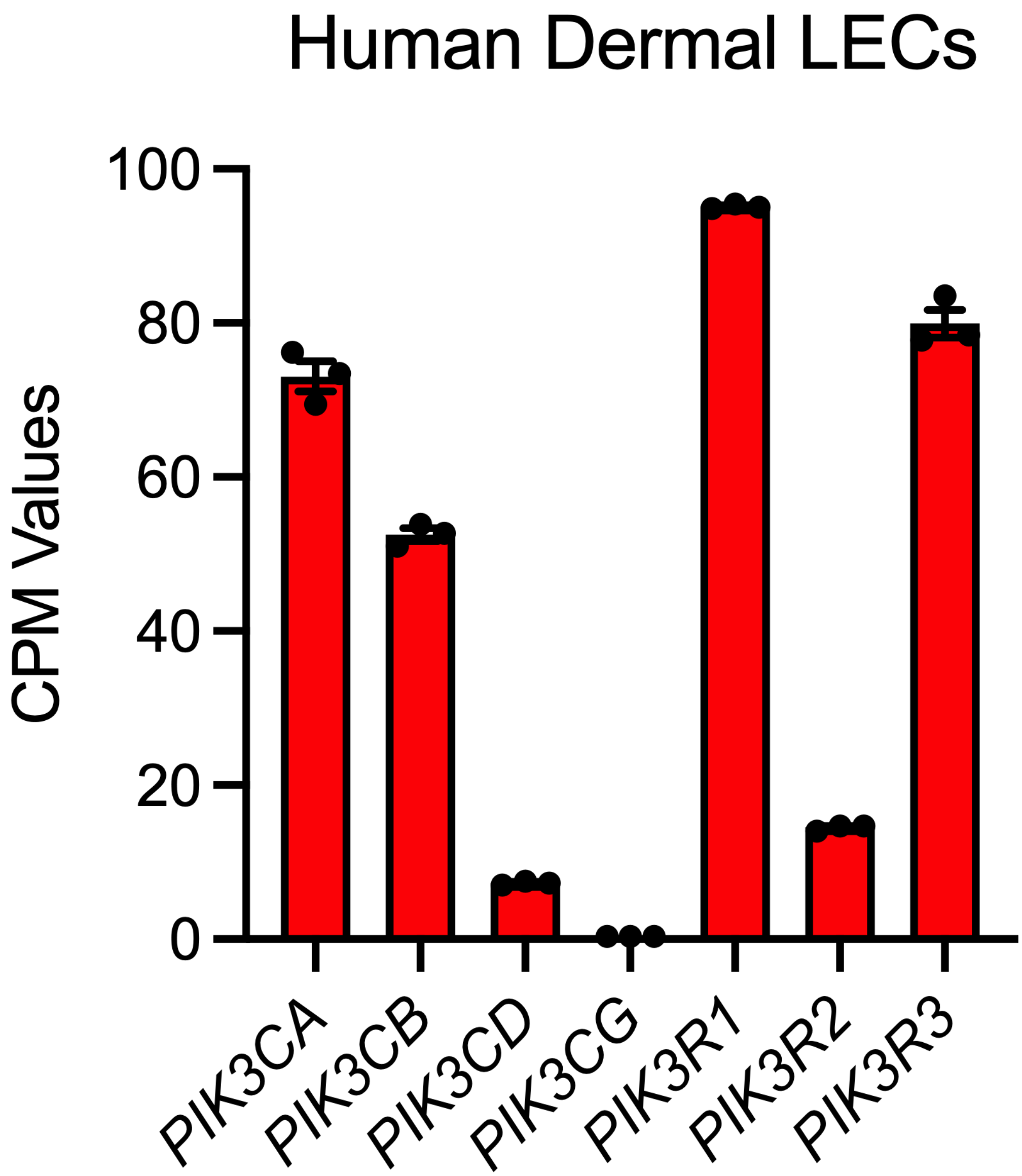

Fig. S1. Graph showing counts per million (CPM) values for PI3K class 1 isoforms in cultured primary human dermal lymphatic endothelial cells.

Disease Models & Mechanisms • Supplementary information

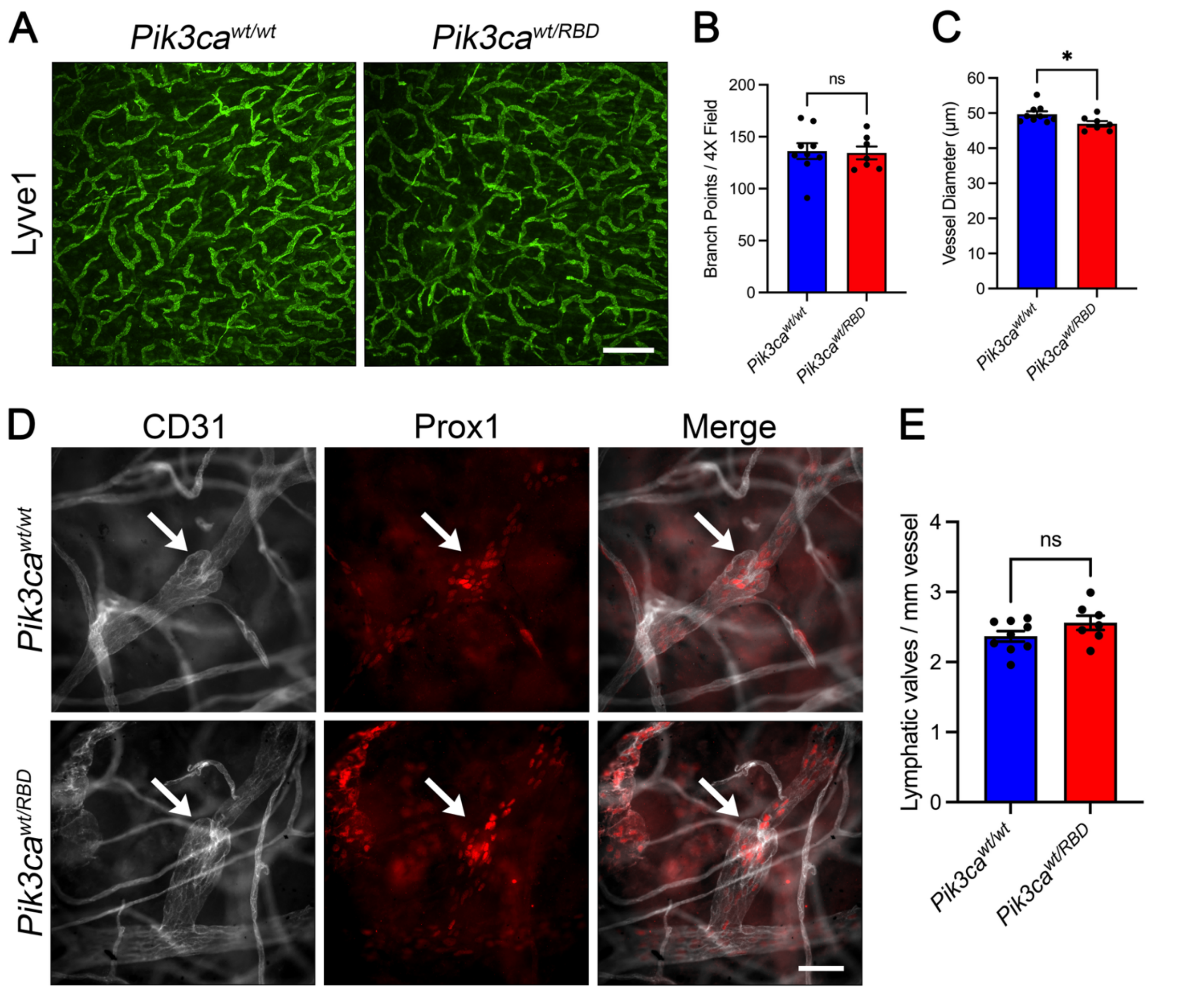

Fig. S2. Lymphatic vessel development is normal in *Pik3ca*$^{wt/RBD}$ mice. A. Representative images of ear skin from three-week-old mice immunostained for Lyve1. **B.** Lymphatic branch points per 4X field in ears from *Pik3ca*$^{wt/wt}$ (136.1 ± 7.5; n = 9 mice) and *Pik3ca*$^{wt/RBD}$ mice (134.4 ± 6.1; n = 7). **C.** Lymphatic diameters in ears from *Pik3ca*$^{wt/wt}$ (49.70 ±0.82; n = 9) and *Pik3ca*$^{wt/RBD}$ mice (46.97 ± 0.77; n = 7). **D.** Representative images of ear skin whole-mounts from three-week-old mice immunostained for CD31 and Prox1. Arrows point to lymphatic valves. **E.** Lymphatic valves per millimeter vessel in ears from *Pik3ca*$^{wt/wt}$ (2.3 ± 0.07; n = 9) and *Pik3ca*$^{wt/RBD}$ mice (2.5 ± 0.102; n = 7). Data are presented as mean ± SEM. n = number of mice. * $P < 0.05$, ns = not significant. Unpaired Student's t-tests (Panels B, C, and E). Scale bar in Panel A = 500 µm. Scale bar in Panel D = 50 µm.

Disease Models & Mechanisms • Supplementary information

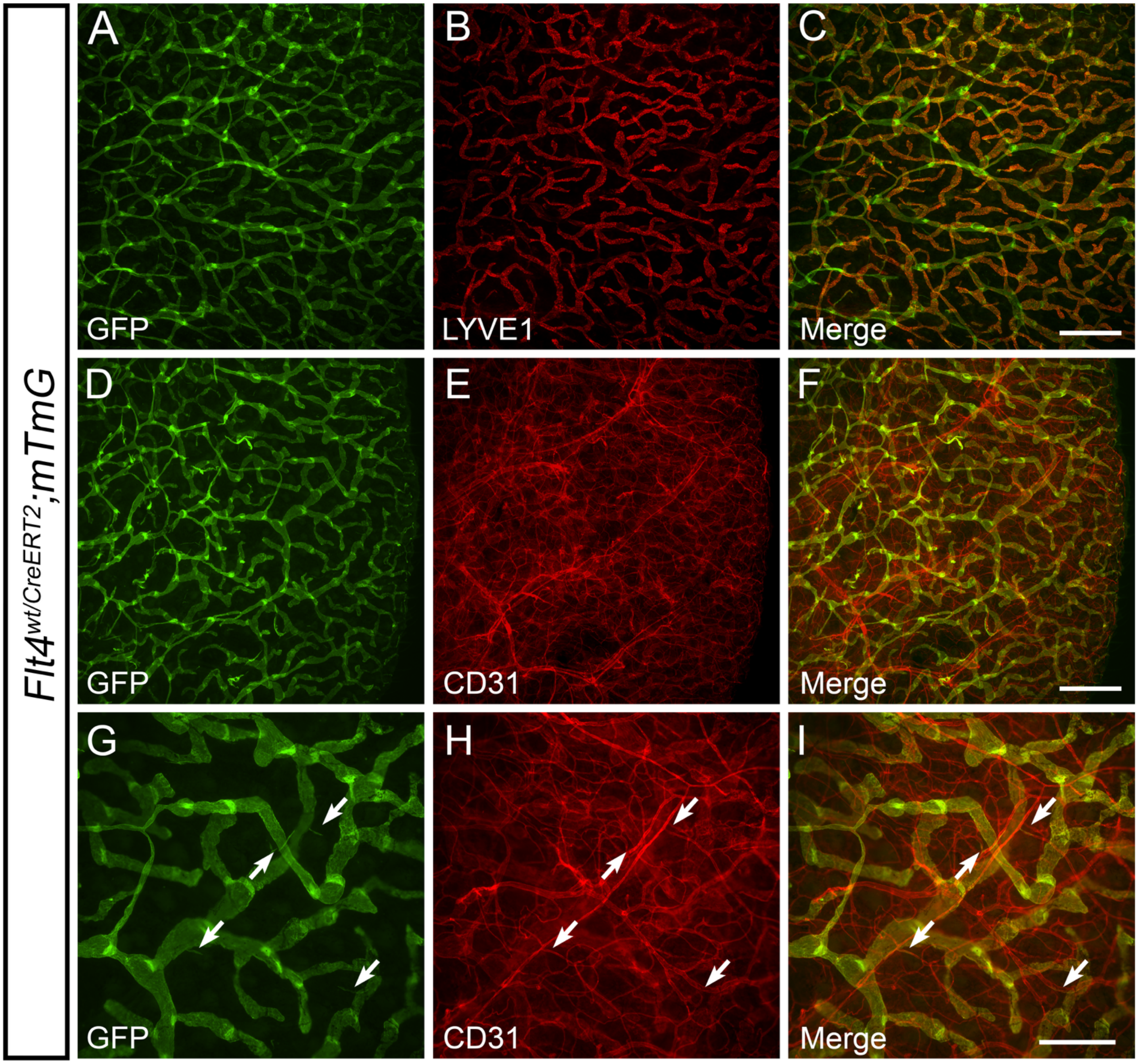

Fig. S3. Lineage-tracing in *Flt4^{CreERT2};mTmG* mice. A-C. Representative images of ear skin from four-week-old mice immunostained for GFP and Lyve1. GFP is widely and specifically expressed in LECs, indicating that our tamoxifen regimen induces widespread and specific recombination in LECs of the ear skin (n = 5 mice). **D-F.** Representative images of ear skin from four-week-old mice immunostained for GFP and CD31. A small fraction of blood vessels expressed GFP (n = 4 mice). **G-I.** Higher magnification of GFP and CD31 staining. Arrows point to GFP-positive blood endothelial cells. Scale bar in C and D = 500 µm. Scale bar in F = 250 µm.

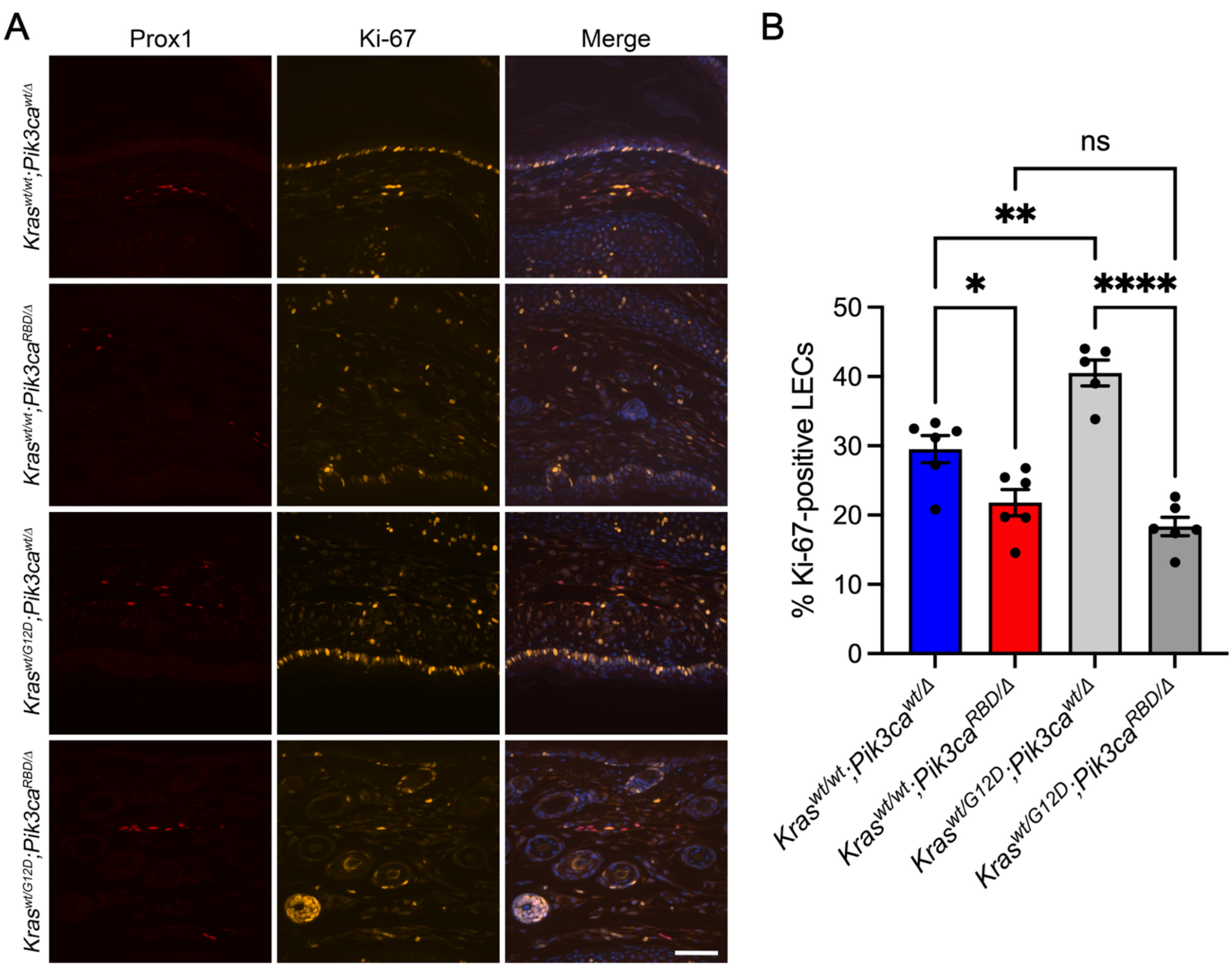

Fig. S4. Blocking RAS activation of p110α reduces Kras^{G12D}-induced LEC proliferation. A. Representative images of ear skin sections stained with antibodies against Ki67 and Prox1. **B.** Graph showing % Ki67-positive LECs in *Flt4$^{wt/CreERT2}$;Kras$^{wt/wt}$;Pik3ca$^{wt/\Delta}$* (29.53 ± 1.94; n = 6), *Flt4$^{wt/CreERT2}$;Kras$^{wt/wt}$;Pik3ca$^{RBD/\Delta}$* (21.80 ± 1.89; n = 6), *Flt4$^{wt/CreERT2}$;Kras$^{wt/G12D}$;Pik3ca$^{wt/\Delta}$* (40.52 ± 1.88; n = 5), and *Flt4$^{wt/CreERT2}$;Kras$^{wt/G12D}$;Pik3ca$^{RBD/\Delta}$* mice (18.36 ± 1.33; n = 6). Data are presented as mean ± SEM. n = number of mice. * $P < 0.05$, ** $P < 0.01$, **** $P < 0.0001$. ANOVA Tukey's multiple comparisons. Scale bar = 50 µm.

Disease Models & Mechanisms • Supplementary information
